# Dietary Patterns, Gut Microbiota and Sports Performance in Athletes: A Narrative Review

**DOI:** 10.3390/nu16111634

**Published:** 2024-05-26

**Authors:** Yonglin Chen, Keer Yang, Mingxin Xu, Yishuo Zhang, Xiquan Weng, Jiaji Luo, Yanshuo Li, Yu-Heng Mao

**Affiliations:** 1 School of Exercise and Health, Guangzhou Sport University, Guangzhou 510500, China; chenyl946@163.com (Y.C.); keryang014@126.com (K.Y.); 18736412692@163.com (Y.Z.); xqweng2003@163.com (X.W.); 13690947595@163.com (J.L.); pattzinian2002@163.com (Y.L.); 2 The Fifth College of Clinical Medicine, Guangzhou University of Chinese Medicine, Guangzhou 510500, China; xumingxin456@126.com; 3 Guangdong Key Laboratory of Human Sports Performance Science, Guangzhou 510500, China

**Keywords:** gut microbiome, dietary pattern, sports performance, athlete

## Abstract

The intestinal tract of humans harbors a dynamic and complex bacterial community known as the gut microbiota, which plays a crucial role in regulating functions such as metabolism and immunity in the human body. Numerous studies conducted in recent decades have also highlighted the significant potential of the gut microbiota in promoting human health. It is widely recognized that training and nutrition strategies are pivotal factors that allow athletes to achieve optimal performance. Consequently, there has been an increasing focus on whether training and dietary patterns influence sports performance through their impact on the gut microbiota. In this review, we aim to present the concept and primary functions of the gut microbiota, explore the relationship between exercise and the gut microbiota, and specifically examine the popular dietary patterns associated with athletes’ sports performance while considering their interaction with the gut microbiota. Finally, we discuss the potential mechanisms by which dietary patterns affect sports performance from a nutritional perspective, aiming to elucidate the intricate interplay among dietary patterns, the gut microbiota, and sports performance. We have found that the precise application of specific dietary patterns (ketogenic diet, plant-based diet, high-protein diet, Mediterranean diet, and high intake of carbohydrate) can improve vascular function and reduce the risk of illness in health promotion, etc., as well as promoting recovery and controlling weight with regard to improving sports performance, etc. In conclusion, although it can be inferred that certain aspects of an athlete’s ability may benefit from specific dietary patterns mediated by the gut microbiota to some extent, further high-quality clinical studies are warranted to substantiate these claims and elucidate the underlying mechanisms.

## 1. Introduction

In recent decades, with the rapid development of competitive sports worldwide, there has been an increasing demand for greater sports performance. Factors such as training strategies, dietary patterns and training environments have garnered significant attention in improving sports performance. Among these factors, dietary patterns are particularly crucial alongside training strategies. It is imperative for athletes to consume adequate nutrition to optimize their condition during training and facilitate proper recovery afterwards [1,2]. Different dietary patterns may yield varying effects on athletes’ sports performance and be suitable for different athletic specialties [3,4,5]. However, there is a paucity of comprehensive reviews examining the potential mechanisms by which dietary patterns influence sports performance.

The human intestinal tract harbors a dynamic and complex bacterial community known as the gut microbiota, which emerging evidence suggests has beneficial effects on human health, including strengthening the gastrointestinal barrier, improving immune function, and regulating glucose and fat metabolism [6]. Consequently, there is growing interest in investigating whether the gut microbiota acts as a mediator for various diseases such as obesity, diabetes, cardiovascular diseases, and non-alcoholic fatty liver disease (NAFLD) [7]. Furthermore, recent attention has focused on exploring the potential role of the gut microbiota as a mediator between dietary patterns, especially for specific micronutrients such as dietary fiber and anthocyanins (ACNs) that are abundant in dietary patterns, and sports performance in athletes [8,9,10]. However, most studies have primarily evaluated the effects of supplements, like probiotics, on athletic performance rather than deeply investigating the relationship between dietary patterns and sports performance through their impact on the gut microbiota. This may be attributed to the complex interaction among different nutrients within dietary patterns and a limited scientific understanding of their specific influence on sports performance. Moreover, the application of dietary patterns on animal models may pose challenges, while using human models may impede the exploration of the potential mechanisms. Therefore, this review aims to summarize recent studies examining how some primary dietary patterns affect sports performance in athletes while also proposing some possible mechanisms involving nutrient-mediated interactions with the gut microbiota to provide practitioners with insights into enhancing sports performance through targeted dietary patterns.

## 2. The Overview of Gut Microbiota

### 2.1. Gut Microbiota

The human microbiota is defined as the microorganisms that exist in symbiosis with the human body, encompassing approximately 10^14^–10^15^ bacteria [11]. It comprises bacteria, archaea, fungi, viruses, bacteriophages, and protozoa [12]. These microorganisms colonize various regions of the human body from birth onwards and are predominantly concentrated in the oral and nasal cavities, skin, the urogenital tract and the gastrointestinal tract [11]. Notably, within the gastrointestinal tract, recent studies have revealed a microbial cell count comparable to that of host cells [13]. Among the vast array of bacterial cells constituting the gut microbiota, which comprises around 2000 identified species [14,15,16], the microbial concentration gradually increases along the gastrointestinal tract, with an abundance of particular anaerobic taxa [17,18]. In the stomach, the acidic pH limits the existence of bacteria, so it presents the lowest number of bacteria, which are primary represented by *Lactobacillus*, *Candida*, *Streptococcus*, and *Helicobacter pylori*. However, in the colon, the favorable pH creates a more suitable habitat for bacteria such as *Bacteroides*, *Clostridium*, *Bifidobacterium*, and *Enterobacteriaceae*, and most of these species are obligate anaerobic bacteria, which participate in the decomposition of polysaccharides and the production of short-chain fatty acids (SCFAs) [19].

The gut microbial composition and diversity undergo changes with aging and are influenced by various factors. For instance, the mode of delivery significantly impacts the initial colonization of bacteria. It has been suggested that infants born through natural delivery predominantly harbor *Lactobacillus* and *Prevotella* species in their gut microbiota, while those born via Cesarean section tend to possess microbiota dominated by *Streptococcus*, *Propionobacterium*, and *Corynebacterium* bacteria. In adulthood, the gut microbiota forms a relatively stable community, but it might vary among individuals. This microbiota community is mainly represented by the *Bacteroidota* and *bacillota* phyla, as well as *Escherichia* and *Lactobacillus* to a lesser extent, but *Bifidobacterium* species remains constant. Among the elderly, Bifidobacterium species decrease in quantity, but Escherichia and *Lactobacillus* tend to increase [20,21]. Apart from the delivery mode, numerous other factors can also influence the diversity of the gut microbiota, including dietary habits, antibiotic usage, host genetics, lifestyle choices, surgical interventions, substance abuse disorders, mental health conditions, and physical exercise [6,7,19,22].

### 2.2. The Main Function of Gut Microbiota on Health

For a considerable duration, extensive research has focused on the perspective that bacteria are pathogenic to humans, exemplified by *Streptococcus pyogenes*, *Bordetella pertussis*, *Corynebacterium diphtheriae*, *Clostridium tetani*, *Salmonella typhimurium*, *Vibrio cholera*, and numerous others [22,23,24,25]. However, the majority of the microbiota are non-pathogenic and even crucial for human health. Substantial evidence now suggests that the gut microbiota plays a pivotal role in human well-being. It participates in metabolic functions by processing indigestible dietary residues and producing SCFAs, which contribute to host metabolic homeostasis [26]. SCFAs subsequently influence mucosal or systemic circulation to impact peripheral organs and tissues. Apart from SCFAs, numerous other microbial metabolites also play crucial roles in various physiological functions. These include bile acids, which promote lipid uptake and maintain gastrointestinal function; lipids such as Lipopolysaccharide (LPS) and Peptidoglycan, which enhance immune system function and regulate glucose homeostasis through the activation of the brain–enteric–liver axis; and choline, which regulates lipid metabolism and glucose homeostasis [27,28,29]. The bacteria species of the gut microbiota also participate in the synthesis of glycans, amino acids, vitamins and other essential components of the human metabolism [14,30]. Furthermore, the gut microbiota actively contributes to fortifying the gastrointestinal barrier by promoting the proliferation and turnover of epithelial cells, thereby enhancing its physiological function. Toll-like receptors (TLRs) play a key role in this process [30,31,32,33]. Within the small intestine’s epithelium cells, Paneth cells recognize the enteric bacteria and subsequently initiate the expression of diverse antimicrobial factors through TLR activation, effectively safeguarding against pathogenic bacterial infiltration [31,33,34]. Additionally, the microbiota stimulates immunoglobulin (IgA) secretion and the production of antimicrobial molecules that inhibit the proliferation and colonization of pathogenic bacteria, thus facilitating the development of gut-associated lymphatic tissue (GALT) and bolstering the host immune system [34,35]. The immune system detects pathogen-associated molecular patterns (PAMPs), which are TLR ligands, enabling it to identify potentially pathogenic bacteria, and consequently leading to increased cytokine levels and the enhanced activation of T cells against these pathogens as a response. Although the gut microbiota has many benefits for the human body, dysbiosis characterized by a quantitative and qualitative imbalance in the microbial composition, along with reduced diversity among species, can give rise to various disorders, including diabetes, cardiovascular diseases, inflammatory bowel diseases (IBD), NAFLD, and obesity. Notably, the presence of *Akkermansia muciniphila*, which represents 3–5% of the typical intestinal microbial members, is decreased in obese people, and *Alistipes putredinis*, which belongs to the phylum Bacteroidota, seems to be represented in people with type 2 diabetes and obesity [19,36,37,38,39]. In this context, several studies have demonstrated that dietary interventions as well as exercise interventions hold promise as effective strategies for modifying the composition and diversity of the gut microbiota towards a more favorable community structure [19,40,41,42,43].

## 3. The Relation between Gut Microbiota and Exercise

**Figure 1:**
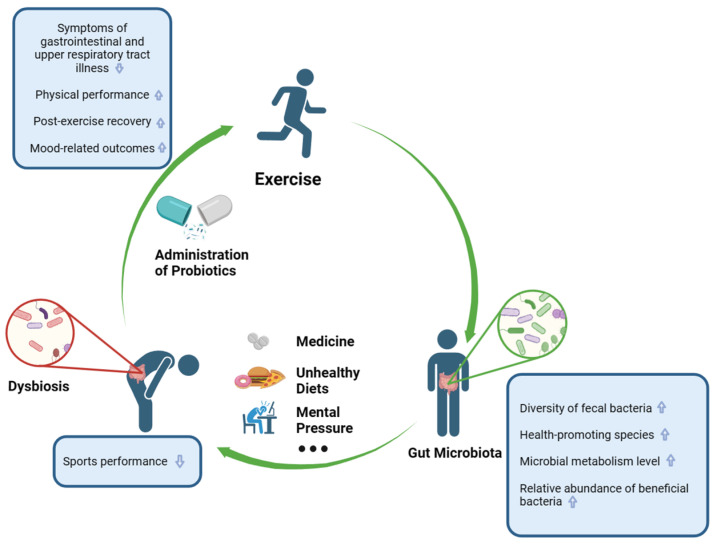
The interaction between exercise and the gut microbiota. Exercise can lead to changes in the gut microbiota [44,47,48,49,50,51]. Unhealthy lifestyles can lead to dysbiosis [36,39]. The administration of probiotics can affect the condition of the gut microbiota, which can subsequently affect sport performance [6,52]. The upward arrows indicate a rise or improvement, the down arrows indicate a drop.

Exercise is widely acknowledged to have a positive impact on human health, and recent studies have increasingly focused on its relationship with the gut microbiota (Figure 1). In contract to sedentary subjects, athletes and physically active individuals exhibit a greater diversity of fecal bacteria, an abundance of beneficial species [44,45,46], and a heightened microbial metabolism, as evidenced by increased activity in the carbohydrate and amino acid metabolic pathway [45,46,47]. Moreover, regular endurance exercise modulates the composition of the gut microbiota and reduces the presence of inflammation-associated proteobacteria [19].

### 3.1. Gut Microbiota in Athletes

A growing body of research has demonstrated that exercise exerts a modulatory effect on the gut microbiota, leading to a distinction in the microbial composition between athletes or physically active individuals and sedentary counterparts. As depicted in Table 1, there were significant differences in the major taxa at various levels between the two population groups. It is worth noting that the trend observed in the Bacteroidetes to Firmicutes ratio between the two groups across different studies was inconsistent [53,54], which may be attributed to several factors including substantial individual variance, human species, and enterotypes [55].

But generally, it is widely accepted that athletes exhibit an enrichment of health-promoting species within their gut microbiota, such as a higher abundance of *Akkermansia* spp. and *Prevotella* spp. [44,47,48,49,53,54,55]. The study conducted by Clarke et al. on male international rugby players from Ireland investigated the dietary intake and physical activity of these athletes, revealing a higher α-diversity in the gut microbiota compared to sedentary controls [44]. The study also included two sedentary control groups consisting of healthy non-professional athletes with different a body mass index (BMI), including a high BMI (BMI > 28) and low BMI (BMI < 25). According to the findings, the professional athletes exhibited greater diversity in their fecal microbiota compared to both control groups. The gut microbiota of elite athletes consisted of 22 phyla of bacteria, while only 11 and 9 phyla were found in the low and high BMI groups, respectively. Notably, increased *Akkermansia muciniphila*, associated with the lean phenotype, was observed in professional athletes and the low BMI group compared with the high BMI group. *Akkermansia muciniphila*, associated with positive metabolic function, is a mucin-degrading bacterium that inhabits the nutrient-rich mucus layer of the gut [56]. Furthermore, this study suggested that the microbial metabolism levels differed between professional athletes and sedentary groups, as indicated by the increased activity in the carbohydrate and amino acid metabolism pathways in athletes. However, it is important to note that the differences in dietary patterns, which refer to a higher total energy, macronutrient (especially protein), and fiber intake in professional athletes compared with the control group, may also influence the gut microbial composition [44].

Additionally, a study revealed that competitive cyclists exhibited a decreased relative abundance of *Bacteroides* spp. Furthermore, the relative abundance of *Prevotella* spp. was found to be higher in cyclists who engaged in training for more than 11 h per week compared with those who trained less frequently [45]. These findings provide evidence supporting the notion that physical exercise can induce alterations in the composition of the gut microbiota.

### 3.2. Impact of Exercise Interventions on Gut Microbiota

To further substantiate the impact of physical exercise intervention on the gut microbiota, several studies have been conducted to explore the causal relationship between exercise and alterations in the gut microbial composition (Figure 1). One study demonstrated that an endurance exercise intervention induced modifications in the gut microbial composition of sedentary, non-trained Finnish women, while controlling for factors such as dietary habits, weight, and body composition [50]. Notably, there were no significant changes observed in the total energy intake or macronutrient and dietary fiber consumption following training. Moreover, no discernible differences were found in the α-diversity of the gut microbiota or the phylum-level relative abundance between pre-intervention and post-intervention samples. However, endurance exercise did lead to an increase in the relative abundance of members of the genera *Verrucomicrobia* and *Akkermansia*, while reducing the levels of inflammation-associated Proteobacteria within the gut.

In addition to endurance exercise, resistance training also exerts an influence on the gut microbiota. Smith et al. demonstrated that 10 weeks of resistance training can improve the alpha diversity in younger untrained adults [57]. Another study conducted by Dupuit et al. explored the impact of a combination of high-intensity interval training (HIIT) and resistance training on the gut microbiota of postmenopausal women [58]; the authors indicated that the training intervention did not significantly change the alpha diversity and overall taxonomy of the fecal microbiota but modified the beta diversity, which is inconsistent with the previous study, showing that more research about resistance training is needed.

However, several other factors may also impact the effectiveness of exercise on the gut microbiota. For instance, one study has indicated that BMI could potentially influence the response of the gut microbiota to physical exercise. According to this particular study [59], individuals with different body compositions (lean and obese) exhibit distinct baseline gut microbiota profiles. However, after a 6-week aerobic exercise intervention, no significant difference in the microbiota community composition was observed between lean and obese subjects.

### 3.3. The Influence of Gut Microbiota on Sports Performance

Exercise exerts a significant impact on the composition of the gut microbiota, while it is reciprocally influenced by the gut microbiota. Determining the precise effects of the gut microbiota on sports performance in human clinical studies poses a challenge due to the intricate interplay of nutritional, genetic and environmental factors [6]. However, germ-free animal models provide a novel approach and have already been employed to elucidate the impact of the gut microbiota on sports performance [60].

A cross-sectional study conducted by Hsu et al. investigated the swimming capacity of specific pathogen-free (SPF), germ-free (GF), and *Bacteroides fragilis* gnotobiotic mice. The results revealed that the swim-to-exhaustion time was the longest for SPF mice and the shortest for GF mice, indicating a compromised sports performance in the absence of a gut microbiota [60]. Although the effects of probiotics supplementation have been studied in athletes and physically active populations, the small number of participants, the different exercise intervention programs implemented, and the different training histories of the participants may have influenced the outcomes [61]; therefore, the results remain controversial. However, a review conducted by Marttinen et al. as summarized several benefits of probiotics for the athlete. The authors demonstrated that the administration of probiotics might reduce symptoms of gastrointestinal and upper respiratory tract illnesses, enhance physical performance, improve post-exercise recovery, and improve mood-related outcomes [6,62,63,64,65]. Therefore, there exists a significant association between the composition of the gut microbiota and sports performance (Figure 1).

## 4. The Influence of Several Typical Dietary Patterns on the Gut Microbiota

**Figure 2:**
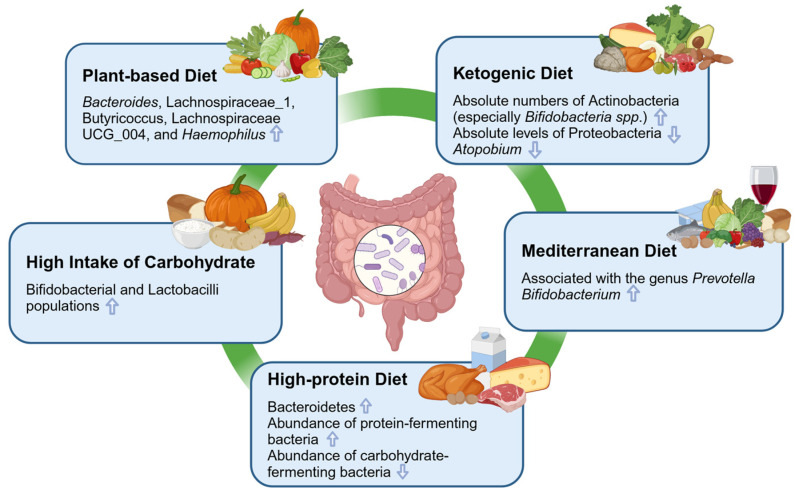
The effects of dietary patterns on the gut microbiota. Different dietary patterns will lead to different changes in the abundance of the gut microbiota. Reference: ketogenic diet [52], plant-based diet [76], high-protein diet [48,77], Mediterranean diet [78,79,80], high intake of carbohydrates [81]. The upward arrows indicate a rise or improvement, the down arrows indicate a drop.

Personal dietary habits play important roles in shaping the composition of the gut microbiota in humans. Although further research is needed to fully understand the intricate relationship between diet and the gut microbiota, numerous studies have highlighted the significant impact of different types of dietary patterns on the composition of the gut microbiota within 24 h [66,67]. The dietary patterns of individuals can be broadly categorized into vegetarians, meat eaters and balanced eaters, each exhibiting a distinct profile in the gut microbiota. Different types of dietary patterns elicit distinct alterations in the proportions of Firmicutes, Bacteroidetes, Proteobacteria and Actinobacteria. Changes in the gut microbiota induced by dietary interventions are observed within 24 h and return to baseline levels within 48 h after discontinuation [66]. These changes encompass alterations in carbohydrate and protein fermentation, intestinal inflammation, fat oxidation, as well as an increase in amino acid availability, potentially promoting protein anabolism [46,68,69,70,71]. Furthermore, the quality, quantity and molecular characterization of carbohydrates, protein, and fat are key factors influencing both the composition and metabolism of the gut microbiota. Unhealthy dietary patterns can stimulate the proliferation of detrimental gut bacteria that pose risks to human health. However, a healthy dietary pattern has restorative effects on beneficial gut bacteria [72]. The maintenance and modulation of beneficial gut microbiota are vital for host health. In addition to general dietary patterns, probiotics supplementation and wholefood supplementation are also common nutrition strategies. Notably, probiotics are defined as living organisms with beneficial effects on health. Most probiotic supplementations contain high concentrations of *Lactobacillus* or *Bifidobacterium* spp., which can support the immune system of the host, regulate gut permeability, and produce sanatory metabolites [73]. Unlike synthetic supplements, wholefood supplements are based on the core idea of supplying the body with nutrients in their pure, unaltered state. This implies that these supplements are rich in a broad spectrum of vitamins, minerals, antioxidants, and other crucial nutrients that are inherently found in the foods from which they are sourced [74,75]. In this section, the influence of several typical dietary patterns on the gut microbiota will be discussed in detail (Figure 2).

### 4.1. Ketogenic Diet

The ketogenic diet (KD) is characterized by a high fat content, a low carbohydrate intake, and an appropriate proportion of protein and other essential nutrients. There are four main types of ketogenic diets: (1) the classical KD with a macronutrient ratio of 4% carbohydrate, 90% fat and 6% protein, (2) medium-chain triglyceride with a macronutrient ratio of 20% carbohydrate, 10% long-chain triglycerides fat, 60% medium-chain triglycerides fat and 10% protein, (3) modified Atkins with a macronutrient ratio of 10% carbohydrate, 65% fat and 25% protein, (4) low-glycemic-index diet with a macronutrient ratio of 10% carbohydrate, 60% fat and 30% protein [82]. It can be seen that the KD does not have a fixed nutrient ratio, but a high proportion of fat and a low proportion of carbohydrates should be guaranteed. The primary objective of this dietary pattern is to shift the glucose metabolism towards fat metabolism through the restriction of carbohydrate intake. Consequently, the KD can effectively lower blood sugar levels and increase free fatty acid and ketone production, thereby influencing neuronal excitability [83]. Notably, the KD is characterized by the production of ketone bodies (3-hydroxybutyrate, acetate and acetoacetate). The elevation in ketones contributes to an increase in anti-inflammatory and antioxidant activity, immune regulation, intestinal mobility and barrier function, cellular growth and differentiation, ionic absorption, as well as the prevention of distal ulcers, Crohn’s disease, and colon cancers. Additionally, the KD was initially employed for managing refractory epilepsy and has progressively extended its application to encompass other neurological disorders [84], such as Parkinson’s disease and Alzheimer’s diseases. With the advancement of medical technology and sports science, there are several studies that have demonstrated the potential of this dietary pattern in enhancing sports performance in some ways [85,86,87]. Nevertheless, this diet still has some limitations; for example, the ability of muscle to use glycogen for oxidation is impaired after long-term ketoadaptation, leading to an inability to utilize the available glycogen, which provides a more effective energy source when the oxygen supply becomes limiting. Therefore, the performance of higher-intensity endurance exercise will be limited, which might increase the risk of injury for athletes [88].

A study conducted by Ang et al. in both mice and humans demonstrated that the ketogenic diet resulted in decreased levels of *Bifidobacterium*, which was mediated by the increased production of ketone bodies, especially beta-hydroxy butyrate. The decrease in *Bifidobacterium* reduced the levels of intestinal and visceral fat pro-inflammatory Th17 cells, which might be a potential mechanism contributing to the ketogenic diet’s ability to reduce body fat because of the relationships between obesity and chronic low-grade inflammation. Furthermore, the ketogenic diet also decreased *Lactobacilli* and increased *Fusobacteria* and *Escherichia* [89].

Several studies have indicated that variations in the quantity and source of dietary fat can exert distinct effects on the host, with some of these effects potentially mediated by the gut microbiota. The consumption of saturated fat has been shown to increase the abundance of bacteria expressing LPS, leading to elevated levels of LPS and a pro-inflammatory state known as metabolic endotoxemia. Furthermore, excessive fat intake is also associated with reduced levels of butyric acid and retinoic acid [90], both crucial for maintaining gut homeostasis. Furthermore, the consumption of saturated fat can enhance the relative abundance of *Bilophila wadsworthia* by facilitating the conjunction of taurine with host LPS, which serves as a terminal electron acceptor and subsequently leads to the production of hydrogen sulfide and secondary bile acids. This cascade may ultimately result in intestinal barrier disruption and immune cell infiltration [91]. Hence, it implies that a KD characterized by a high saturated fat intake could potentially elevate the inflammatory level of the host. Conversely, polyunsaturated fat acid (w-3) can increase SCFAs and promote gastrointestinal integrity and inflammation. Furthermore, polyunsaturated fat increases the abundance of *Bifidobacterium*, *Lactobacilli*, and *Akkermansia muciniphila*, which are also increased by exercise. Thus, polyunsaturated fat might contribute to health and sports performance by mimicking the effects of exercise, but the dose remains controversial; more research is needed to investigate this [92,93].

Notably, with the advancement of research on diet and nutrition, the classical KD has undergone certain variations; for instance, the very-low-calorie KD (VLCKD) is characterized by a caloric intake of below 800 kcal/day. One study revealed that the VLCKD results in a more substantial weight reduction, rendering it an excellent option for weight loss [94,95]. Regarding the gut microbiota, a review has summarized the effects of the VLCKD on the gut microbiota [96]; in this study, the authors demonstrated that the abundance of Bacteroidetes, Firmicutes, Proteobacteria, and Verrucomicrobiota in people who undertook the VLCKD increased and that the abundance of Firmicutes, Firmicutes/Bacteroidetes ratio, Proteobacteria, and Actinobacteria decreased. These seemingly contradictory results suggest that further research is warranted to explore the impact of the VLCKD on the gut microbiota. Furthermore, the application of the VLCKD still remains controversial, especially for athletes, because this diet is used more in obese patients. However, the VLCKD could be used to control weight acutely in special sports during a special period with strict medical supervision, such as in gymnastics [97,98,99].

In conclusion, the impacts of the KD on the gut microbiota remains inconclusive and controversial, necessitating further studies to comprehensively understand its effects.

### 4.2. Plant-Based Diet

The plant-based diet is a dietary pattern primarily based on a diverse range of plants, encompassing seeds, fruits, and plant tissues that provide energy for human consumption. This includes cereals, tubers, legumes and their derivatives, as well as fruit and vegetable products. The distinguishing features of the plant-based diet are its high carbohydrate content, low energy density, low fat content, and absence of cholesterol, antibiotics or hormones [100]. Long-term adherence to a plant-based diet not only reduces the risk of many chronic diseases, but also contributes to the lower emission of greenhouse gases such as carbon dioxide during food processing compared to other methods [101]. Consequently, it plays an integral role in promoting human health and environmental preservation [102].

One study explored the microbial composition of 258 participants who adhered to one of four dietary patterns: the Western diet group, flexitarian group, vegetarian group, and vegan group [76]. Notably, the Western food group is characterized by a high intake of energy, salt, saturated fat, simple or added sugar, and a low intake of fruits and vegetables [103]. The vegetarian group is characterized by omitting defined food groups such as meat, sausage, fish, etc., and the vegan group is characterized by additionally omitting dairy products and honey [104,105,106]. Flexitarians generally consume meat or sausage once or twice per week [107]. The western diet group exhibited the lowest abundance of *Bacteroides*, Lachnospiraceae_1, Butyricoccus, Lachnospiraceae UCG_004, and *Haemophilus*; whereas the vegan group showed the highest abundance. For *Dorea*, the *Ruminococcus torques* group, *Eubacterium ruminantium* group, Ruminococcaceae, Lachnospiraceae_2, *Lactobacillus*, and *Senegalimassilia*, the lowest abundance was observed in the vegan group, while the highest abundance was observed in the Western diet group [76]. Notably, a high abundance of Lachnospiraceae in the vegan group indicates the extensive fermentation of plant-based polysaccharides into SCFAs like butyrate, which is beneficial for human health. For example, it serves as a crucial energy source for colonic epithelial cells, regulates intestinal inflammation, and confers protection against colon cancer in humans [76,108,109].

Furthermore, the vegan diet might also contribute to improving performance and promoting recovery in endurance sport by affecting body composition, blood flow, antioxidant capacity, systemic inflammation, and glycogen storage [110].

### 4.3. High-Protein Diet

People who stick to a high-protein diet can take in higher-quality protein and provide the body with amino acids. Protein is a macronutrient, as well as the main component of skeletal muscle. The uptake and catabolism of specific proteins by the liver and skeletal muscle are different, as is their ability to regulate the muscle protein synthetic response [111]. Amino acids can be metabolized into branched-chain fatty acids and SCFAs, ammonia, sulfides, indole, and phenolic compounds via the gut microbiota [112]. Some of these (e.g., SCFAs and indole) may be beneficial for the health of the gut, while other metabolites (e.g., ammonia and p-cresol) may decrease gut epithelium integrity [113].

The high-protein diet is widely popular and frequently adopted by fitness enthusiasts and athletes, particularly for the latter who engage in intense exercise routines that necessitate strict dietary practices to support optimal performance [114]. In contrast to the general population, athletes often consume significantly higher amounts of protein; however, if this excess protein remains unabsorbed, according to a study conducted by Moreno-Pérez et al. [48], it can enter the colon and promote the growth and selection of specific bacteria. In this study, a 10-week supplementation with protein, commonly used to meet the elevated protein requirements among individuals undergoing training, resulted in an increased abundance of Bacteroidetes while decreasing the taxa associated with overall health, including *Roseburia* spp., *Blautia* spp., and *Bifidobacterium longum*, among runners. Another study has compared the gut microbiota of bodybuilders consuming a high-protein diet with sedentary controls [77], and found that excessive protein intake increased the abundance of protein-fermenting bacteria such as *Clostridium*, *Bacillus*, *Staphylococcus*, and other species belonging to the Proteobacteria family. Moreover, the high-protein diet might lead to a reduction in carbohydrate-fermenting bacteria, such as *Bacteroides*, *Lactobacillus*, *Bifidobacterium*, *Prevotella*, *Ruminococcus, Roseburia*, and *Faecalibacterium*. The fermentation of incompletely digested protein in the colon might lead to the production of toxic metabolites such as ammonia, biogenic amines, indole compounds, and phenols. However, there was no significant difference in the abundance of selected bacteria (*Bifidobacterium* spp., *Bacteroides* spp., *Faecalibacterium prausnitzii*, *Akkermansia Muciniphila*) between the bodybuilder group and control group; the possible reason for this is that both of the two groups met the criteria for the recommended fiber intake, and the effect of high protein intake on the gut microbiota might have been attenuated by the appropriate intake of carbohydrate and fiber. Therefore, it is imperative to strictly control not only the types of protein consumed, but also the quantity ingested by athletes.

### 4.4. Mediterranean Diet

The Mediterranean diet (MD) originates from the Mediterranean region, including Greece, Spain, France, and Italy. It is based on the traditional dietary habits of the countries bordering the Mediterranean Sea. This dietary habit is characterized by a high intake of fruits, vegetables, cereals, olive oil, legumes and tree nuts, a moderate intake of seafood, and a low intake of sugar sweetened foods, red and proceed meat, and carbonated beverages [4]. However, there remains controversy surrounding its precise definition. In a recent study [115], the authors attempted to establish a unified definition of the MD by considering daily servings of key foods and their nutrient content: Vegetables: 3 to 9 servings; Fruit: 0.5 to 2 servings; Cereals: 1 to 13 servings; Olive oil: up to 8 servings.

Considering its energy intake and macronutrient composition, the MD can be classified as a predominantly plant-based dietary pattern, encompassing vegetables, fruits, cereals, and olive oil [116,117,118]. It is notable that the MD exhibits a relatively high fat content, with monounsaturated fats comprising twice the amount of saturated fat. The primary source of monounsaturated fats in the MD is olive oil, which is closely associated with the traditional olive cultivation in the Mediterranean region. Additionally, the MD allows for the moderate consumption of white and red meat. Extensive evidence supports that adherence to the MD promotes longevity while reducing the metabolic risks associated with diabetes mellitus, obesity, and other metabolic syndromes [119,120]. Moreover, it demonstrates a reduced risk of malignancy and cardiovascular disease while enhancing cognitive function [116].

Numerous studies have consistently demonstrated that the gut microbiota plays a crucial role as a potential mediator in the association between the MD and human health. A study has indicated that nearly 60% of the overall composition of the gut microbiota is responsive to dietary changes [121]. The MD can not only modulate the diversity and composition of the gut microbiota, but also improve the generation of SCFAs due to its high proportion of plant-based food [78]. Previous research has shown an association between the MD and *Prevotella* [78,79], while another study suggests that the MD contributes to reducing dysbiosis and increasing *Bifidobacterium* among patients with metabolic syndrome [78,80]. However, it should be noted that not all studies support the positive influence of the MD on the gut microbiota. Some investigations found no significant difference in the gut microbiota composition between individuals adhering to either the MD or Western diet interventions for 6 months. Therefore, further research is warranted to comprehensively explore the impact of the MD on the gut microbiota [78,122]. Furthermore, according to a narrative review conducted by Griffiths et al., the application of MD or individual foods and compounds in this dietary pattern might have potential positive effects on oxidative stress, inflammation, injury, illness risk, and cognitive and vascular function in competitive athletes [4].

### 4.5. High Intake of Carbohydrate

Limited research has been conducted on the high-carbohydrate diet, probably due to the fact that a high intake of carbohydrate is not typically considered as an independent dietary pattern but rather as a supplementary measure in other dietary patterns to meet the energy requirements of athletes, given its role as a primary fuel source during exercise [123]. It is recommended that athletes consume ample amounts of simple carbohydrates to maintain glucose homeostasis and limit their fiber intake prior to exercise in order to minimize gastrointestinal discomfort. Non-digestible carbohydrates will be discussed later. Adequate carbohydrate consumption is crucial for athletes. The ingestion of simple carbohydrates before and during exercise (e.g., glucose, fructose, sucrose) can alleviate fatigue, facilitate rehydration and the maintenance of optimal fluid balance, and enhance sports performance [124,125,126,127,128]. For example, lactose may serve as an effective fuel source before, during and after exercise, thereby enhancing sports performance and aiding recovery while also potentially exerting beneficial effects on the gut microbiota, such as increasing Bifidobacteria and Lactobacilli populations [81]. According to a study conducted by Faits et al., which discusses the different effects of simple, refined, and unrefined carbohydrate-containing foods on the gut microbiota, after the consumption of an unrefined carbohydrate diet, the abundance of *Roseburia* was higher and fecal secondary bile acid concentrations were lower relative to the simple carbohydrate diet, whereas the abundance of *Anaerostipes* was higher after the consumption of a simple carbohydrate diet relative to the refined carbohydrate diet [129].

Notably, athletes in many sports often consume a high amount of fast-absorbed carbohydrates to maximize glycogen storage. However, they also aim to avoid non-digestible carbohydrates in order to prevent intestinal issues and other unfavorable syndromes that can negatively impact sports performance, such as bloating and diarrhea [130]. While a high intake of fast-absorbed carbohydrates can increase energy storage during training or competition, a low intake of dietary fiber may lead to the reduced production of short-chain fatty acids (SCFAs), altered intestinal transit times, and a loss of bacterial diversity [3], all of which have negative implications for long-term health [131]. Therefore, it is important for athletes to consume a certain amount of fiber to generate less gas after fermentation by the gut microbiota in order to gain health benefits and avoid gastrointestinal issues.

## 5. Different Dietary Patterns and Sports Performance—Gut Microbiota as the Mediator

### 5.1. Gut Microbiota as the Mediator

With the advancement of competitive sports, whether it pertains to athlete-to-athlete competition or the audience’s heightened expectations for sporting event enjoyment, both lead to elevated demands on athletes’ capabilities. Numerous factors, such as exercise intensity, dietary patterns, lifestyle choices and genetic inheritance, among others, can influence the sports performance of athletes or physically active individuals. The gut microbiota—an integral component of human beings since birth—has emerged as a prominent area of research interest due to its intricate composition and structure. Several studies have indicated disparities between the gut microbiota profiles of athletes and those of normal individuals. Numerous investigations have attempted to establish whether the gut microbiota is a mediator linking dietary patterns and sports performance. Here, we present a concise overview of the current primary evidence pertaining to the aforementioned dietary patterns and the discuss probable mechanisms by which dietary patterns affect sports performance (Table 2).

To date, the impact of the KD on sports performance remains controversial. As mentioned earlier, the consumption of saturated fat increases the LPS level in the host, which activates toll-like receptors 4 (TLR4) and cluster of differentiation 14 (CD14), leading to obesity, increased inflammatory indices in white adipose tissue (WAT), and insulin resistance [133]. Interestingly, this effect was observed only in subjects consuming saturated fat. These findings suggest that athletes implementing a KD can increase their intake of unsaturated fats to avoid inflammation and insulin resistance. Additionally, the VLCKD may have a beneficial effect on obesity by regulating the gut microbiota and restoring homeostasis [96]. The study by Gutierrez-Repiso et al., 2019, discussed the association between the VLCKD and weight loss through the gut microbiota [134]. On one hand, the authors reported that the abundance of *Butyricimonas* and *Oscillospira* increased at the genus level. Notably, *Oscillospira* is positively associated with high-density lipoprotein, butyrate and leanness, while *Butyricimonas* is positively associated with energy metabolism and homeostasis between the microbiota and host. Both of these gut microbiota are beneficial for weight loss. On the other hand, the proportion of *Serratia* and *Citrobacter,* whose abundance has been positively correlated to obesity, decreased. Therefore, the VLCKD can positively regulate the gut microbiota after obesity-relative dysbiosis. This dietary pattern enables rapid short-term weight reduction, making it suitable for athletes who need to quickly regain an optimal weight.

In terms of the high-protein diet, research has primarily focused on the impact of protein. Evidence suggests that the gut microbiota contributes to the absorption and utilization of protein, as well as the anabolism and functionality of skeletal muscle by providing fuel and storage and modulating inflammation. For example, the co-administration of the probiotic *Bacillus coagulans (GBI-30,6086)* with protein has been shown to reduce the inflammation of epithelial cells, enhance nutrient absorption and stimulate protease production for increased amino acid uptake in humans [135,136]. These effects have the potential to mitigate muscle damage and facilitate muscle recovery, thereby promoting sports performance [137]. In addition, animal studies have been conducted to investigate the effects of different protein types on the gut microbiota, with a particular focus on comparing animal-based proteins to plant-based proteins [138,139,140,141]. These studies have demonstrated that the consumption of meat protein leads to a higher abundance of Lactobacilli and an increased ratio of Firmicutes to Bacteroidetes, while also reducing levels of butyrate-producing bacteria (e.g., *Bacteroides* and *Prevotella*), LPS-binding protein, and transcription factor CD14 receptor when compared to non-meat protein intake. Furthermore, dairy proteins appear to have an intermediate effect between meat and non-meat proteins. It is worth noting that LPS-binding protein binds to CD14 in order to activate macrophages, which can subsequently produce inflammatory cytokines, leading to inflammation. Based on these findings, it can be hypothesized that athletes may benefit from consuming more meat protein rather than non-meat protein in order to mitigate muscle inflammation and maintain optimal sports performance. However, the studies mentioned above have primarily focused on rodents, with limited exploration of their effects on humans; one reason for this may be that it is difficult to intervene individually with different types of proteins in humans, and that other nutrients might interfere with the experimental results. A human study investigating the impact of various protein types on gut the microbiota and incorporating a high- or low-saturated fat component into the study design indicated that the intake of saturated fat may cover up the effects of protein types [142]. Another study conducted by Losasso et al. that compared the influence of vegan, vegetarian and omnivore-oriented Westernized dietary styles on the gut microbiota indicated that vegans and vegetarians show higher α-diversity than those who consume animal protein, the main operational taxonomic units associated with the phylum Bacteroidetes, and the genus *Prevotella*, which can improve glycogen storage, was more prevalent among individuals that consume more fiber and vegetable protein. However, the subjects in this study also consumed different nutrients, which may have influenced the results [143]. Consequently, it can be inferred that enhancing the protein bioavailability and absorption, as well as muscle protein synthesis, serves as an important mechanism through which the gut microbiota influences muscle mass and function. This mechanism is likely regulated by SCFA production, thereby affecting insulin sensitivity, inflammation, and insulin growth factor I (IGF-I) release to maintain anabolic–catabolic balance. Furthermore, more studies elucidating the effects of different protein types in humans that consider other dietary components beyond just protein consumption are needed [144,145].

It is noteworthy that dietary fiber plays an essential role in both the plant-based diet and MD, as it constitutes their main component. Dietary fiber is composed of complex carbohydrates, including fermentable (mainly soluble) and non- or poorly fermentable (mainly insoluble) fibers, as well as oligosaccharide. Dietary fiber influences the composition of the gut microbiota, contributing to the establishment and maintenance of a healthy and diverse gut microbiota while improving intestinal immunity [146]. However, the insufficient intake of dietary fiber may have adverse effects on human health. The dietary fiber in the aforementioned dietary patterns includes “Microbiota-accessible carbohydrate (MACs)”, which are complex carbohydrates found in fruits, vegetables, legumes, and whole grains [116]. A study conducted by Xu et al. has shown that a high intake of MACs promotes lipid profile improvement, glycemic control, body weight reduction, and an inflammatory maker decrease compared with low MAC intake [147]. Furthermore, MACs can influence the gut microbiota and modulate the growth of species that produce SCFAs, which are the end products of dietary fiber fermentation in the intestines. SCFAs play an essential role in human metabolism. A study has indicated that SCFAs can directly activate Adenosine 5‘-monophosphate (AMP)-activated protein kinase (AMPK) by increasing the AMP/ATP ratio in skeletal muscle and liver or indirectly activate it via the Ffar2-leptin pathway [148,149,150]. The activation of AMPK triggers the expression of proliferator-activated receptor gamma coactivator PGC-1α, which is known to regulate the transcriptional activity of key factors including peroxisome proliferator-activated receptors PPARα, PPARδ, PPARγ, liver X receptor (LXR), and farnesoid X receptor (FXR). These factors are crucial to regulate the metabolism of cholesterol, lipid, and glucose. The fatty acid oxidation of muscle and liver is ultimately enhanced, while de novo fatty acid synthesis in the liver is reduced [151,152]. In addition, SCFAs have been demonstrated to enhance the protein expression of PGC-1α and uncoupling protein-1 (UCP-1) in brown adipose tissue, subsequently promoting thermogenesis and fatty acid oxidation. These results suggest that the plant-based diet and MD, which are rich in dietary fibers, could be considered for dietary planning among weight-conscious athletes such as marathon runners. However, it is still crucial for endurance athletes to maintain an adequate intake of simple carbohydrates. For instance, in an international marathon competition that typically lasts for a minimum duration of approximately two hours, athletes require sufficient glycogen reserves to optimize their sports performance. Therefore, carbohydrate loading is commonly employed by endurance athletes as a strategy to enhance glycogen concentrations prior to competitions. However, it is crucial to avoid consuming carbohydrates that are indigestible and unabsorbable in the small intestine, such as fiber and resistant starch [88]. Nevertheless, scientific evidence suggests that adopting a high-carbohydrate, low-fiber dietary pattern can have detrimental effects on the gut microbiota and overall health. These effects include disruptions in intestinal transit times, the loss of bacterial diversity, and reduced SCFA production [153,154,155]. Thus, athletes should judiciously manage both the timing and quantity of their intake of simple carbohydrates and nondigestible carbohydrates to optimize their sports performance while minimizing gastrointestinal distress.

Notably, a clinical study conducted by Jang et al. in Korea revealed an inverse correlation between total protein intake and the diversity of the gut microbiota, showing that the athletes in resistance sport who have a high protein diet showed a decrease in SCFs-producing commensal bacteria [49]. However, another study demonstrated a positive correlation between a high protein intake and microbial diversity; the gut microbiota of athletes consisted of 22 phyla of bacteria, while only 11 and 9 phyla were found in the low and high BMI groups [44]. It is worth noting that Korean athletes did not meet the recommended dietary fiber intake (≥25 g/day; median intake in bodybuilders 19 g/day, endurance athletes 17 g/day) [49]. In contrast, Irish rugby players’ dietary fiber intake met the recommendation level (median intake 39 g/day) [44]. Undigested dietary fiber serves as an essential energy and carbon source of gut microbiota, contributing to its diversity and acting as a substate for SCFA synthesis. Therefore, it can be inferred that combining a high-protein diet with low-dietary-fiber diet may have detrimental effects on the gut microbiota composition. This finding suggests that dietary fiber also plays an important role in the high-protein diet. Further investigations are warranted to ascertain whether alterations in SCFA levels serve as a pivotal mediator of the favorable physiological effects associated with a high dietary fiber intake.

In addition to dietary fiber, anthocyanins (ACN) have recently attracted the attention of many researchers. Amongst some of the dietary patterns mentioned above, fruits and vegetables are important components, particularly certain fruits that are abundant in ACN, a subclass of polyphenols responsible for the red–blue–purple pigmentation observed in fruits [156,157]. These bioactive compounds possess potent antioxidant and anti-inflammatory properties that can effectively modulate the secondary cascade associated with exercise-induced muscle damage (EIMD) [10,158,159,160,161]. Delphinidin and cyanidin are the most extensively investigated anthocyanins, which also encompass malvidin, peonidin, petunidin and pelargonidin. These compounds exhibit favorable physiological effects in humans [162]. The bioavailability of ACN in the human intestinal tract is limited, with only a fraction of the dietary intake being digested and absorbed in the small intestine. However, this bioavailability can be enhanced through interactions with the gut microbiota [163]. The sugar moieties of ACN undergo hydrolysis by bacterial enzymes in the colon, leading to the transformation of aglycone forms into a variety of compounds, including protocatechuic acid, vanillic acid and gallic acid [164]. According to a study [132], cyanidin consistently converts into protocatechuic acid, which exhibits multiple protective functions for muscle health, such as reducing oxidative stress, promoting mitochondrial biogenesis, and converting skeletal muscle fibers from type II to type I. These effects on oxidative stress reduction and mitochondrial biogenesis may have potential benefits for athlete recovery. Notably, the conversion of skeletal muscle fiber emerges as a promising research domain, deserving significant attention. In the past, the selection of athletes across various sports has heavily relied on hereditary factors due to the perception that one’s skeletal muscle type is genetically predetermined and difficult to change through training. With advancements in our understanding of the skeletal muscle fiber conversion, as well as potential nutritional strategies, the process of athlete selection may become more adaptable. However, humans still cannot make genetic changes. This means that while a certain genetic hereditary factor, such as alpha actinin-3 gene (ACTN3), plays a decisive role in skeletal muscle fiber conversion, dietary patterns could be utilized as a helpful strategy to improve it [165].

However, the specific bacterial taxa responsible for the transformation of anthocyanins into protocatechuic or gallic acid remain unknown. The bacterial enzymes involved in ACN hydrolysis may be present in several taxa of the genera, such as *Bacteroides*, *Clostridium* and *Eubacterium* [26,163]. Furthermore, different microbiota compositions may be associated with distinct pathways of ACN biotransformation, potentially leading to diverse effects ranging from beneficial to unknown outcomes [166,167], implying that the interaction between ACN and the gut microbiota could vary among individuals. Therefore, further research is needed to investigate individual differences in ACN metabolism and its potential health-promoting effects.

### 5.2. Practical Application

As mentioned earlier, different dietary patterns affect sports performance in different ways. Athletes should choose the appropriate dietary pattern on the basis of their actual situation during training. Athletes who need to control their weight strictly during competition in heavy sports, athletics and gymnastics may consider a ketogenic diet, which would enable them to lose weight in a short time, but this dietary pattern also has limitations; it is not suitable for enhancing strength in weight lifters or high-intensity cyclists, for example [168]. In terms of the plant-based diet, current evidence supports that this diet does not have a significant impact on sports performance, but as mentioned before, the special micronutrients in the plant-based diet have anti-inflammation and antioxidant effects to a certain extent, and it would be friendly to vegan athletes [169]. For athletes who seek to gain muscle mass and strength, such as bodybuilders, the high-protein diet is a good choice, because it is necessary to generate more muscle protein and prevent lean mass losses during the periods that restrict energy intake to promote fat loss [170]. Compared with other dietary patterns, the Mediterranean diet may be more suitable for most athletes; both aerobic and anaerobic athletes can select this dietary pattern, whose strengths are that it is rich in foods that can support high energy demands and that it can provide the antioxidants, essential vitamins and minerals that promote recovery [3]. In practice, these dietary patterns are used alternately or in a certain period of time, because any special dietary patterns used for a long time will cause adverse reactions [3,98].

## 6. Conclusions

In recent decades, it has been increasingly acknowledged that the gut microbiota plays an important role in human health and sports performance. As mentioned earlier, the impact of various dietary patterns on the gut microbiota and their subsequent effects on sports performance may vary. Therefore, further evidence is required to substantiate the relationship between different dietary patterns and their components with the gut microbiota and sports performance. In addition, it should be noted that diet is inseparable from the host; it is challenging to strictly disentangle exercise from daily diet during an experiment, as the individual contributions of each participant are difficult to isolate and assess. To date, there remains a dearth of research investigating the intricate interplay between diet, exercise, and the gut microbiota. Additionally, the responses of the gut microbiota to diet may vary among individuals, indicating that the formulation of diet regimens should shift from standardized diet guidelines to flexible recommendations tailored to individual preference and local customs, and the regular reassessment of these dietary regimens is essential. Moreover, the significance of nutrients or compounds in diets that have traditionally been regarded as non-nutritive cannot be disregarded, necessitating an exploration into whether these nutrients exert their effects independently or synergistically. Future research should focus on personalized nutrition strategies for different populations and the combined effects of different nutrients. The aforementioned findings will contribute to a comprehensive understanding of the intricate interplay among exercise, diet, and human health, which has implications not only for athletes’ well-being but also for that of the general population.

**Key Points**

- The interactions between exercise and the gut microbiota play a role in the sports performance of athletes.
- The ketogenic diet, plant-based diet, high-protein diet, and Mediterranean diet may improve sports performance from different aspects.
- The gut microbiota and its metabolites play an important role in the effects of dietary patterns on sports performance.

## Figures and Tables

**Table 1:** Comparison of the gut microbial composition between athletes/physically active population and non-athletes/sedentary population.

Author, Year	Country	Sample Size, Sex and Age	Main Findings on Gut Microbial Composition	
			Athletes/Physically Active Population	Non-Athletes/Sedentary Population
Xu et al., 2022 [53]	China	n = 66 (males = 36, females = 30), Age: 18–25 years	Bacteroidetes (52.53%) Firmicutes (43.99%) Prevotella (20.88%) Bacteroides (24.96%) Faecalibacterium (6.86%) Megamonas (11.67%)	Bacteroidetes (62.81%) Firmicutes (32.14%) Prevotella (26.81%) Bacteroides (25.01%) Faecalibacterium (10.57%) Megamonas (5.15%)
Humińska-Lisowska et al., 2024 [55]	Poland	n = 52, males Age: 19–24 years	Enterotype: Endurance group: Bacteroides-driven (46.70%) Strength group: Prevotella-driven (50.00%)	Enterotype: Control group: Bacteroides-driven (40.90%) Ruminococcus-driven (40.90%)
Hintikka et al., 2022 [54]	Finland	n = 54 (males = 28, females = 26) Age: Athlete group: 27.1 ± 5.1 years Control group: 27.4 ± 5.6 years	Bacteroidetes (50.40%) Firmicutes (46.00%) Proteobacteria (2.30%) Actinobacteria (0.79%)	Firmicutes (48.30%) Bacteroidetes (46.20%) Proteobacteria (3.36%) Actinobacteria (1.57%)

**Table 2:** The probable mechanism of dietary patterns affecting sports performance.

Author, Year	Dietary Pattern	Substance	Subjects	Pathway	Most Important Findings
(Caesar et al., 2015 [115])	Ketogenic diet	Saturated fat	Male mice	LPS/TLR4 pathway	Increases inflammatory indices in WAT
(Minevich et al., 2015 [119])	High-protein diet	*Bacillus coagulans GBI-30, 6086* Protein	Males (*n* = 11)	Promote the absorb and utilize of protein	Produces proteases which can increase amino acid absorption in humans
(Zhu et al., 2017 [121])	High-protein diet	Animal protein	Male rats (*n* = 32)	Decrease the binding of CD14 and LPS-binding protein	Higher abundance of Lactobacilli Higher ratio of Firmicutes to Bacteroidetes Lower butyrate Lower SCFAs-producing bacteria Lower LPS-binding protein Lower transcription factor CD14 receptor Lower inflammation
(Jäger et al., 2007 [81])	Plant-based diet/ Mediterranean diet	Dietary fiber	C2C12 myotubes Female mice	AMPK/PGC-1α pathway	Enhances fatty acid oxidation of muscle
(Yang et al., 2023 [132])	Plant-based diet/ Mediterranean diet	Anthocyanins	C2C12 myotubes Male mice (*n* = 60)	AMPK signaling pathway	Reduces oxidative stress Promotes mitochondrial biogenesis Converse skeletal muscle fiber

This table shows the mechanisms of the effects of nutrients in different dietary patterns on the gut microbiota. AMPK: adenosine 5-monophosphate-activated protein kinase; CD14: cluster of differentiation 14; LPS: lipopolysaccharide; PGC-1α: proliferator-activated receptor gamma coactivator; SCFAs: short-chain fatty acids; TLR4: toll-like receptors 4; WAT: white adipose tissue.
